# A Robust Direction of Arrival Estimation Method for Uniform Circular Array

**DOI:** 10.3390/s19204427

**Published:** 2019-10-12

**Authors:** Zhengqin Xu, Shiqian Wu, Zhuliang Yu, Xingyu Guang

**Affiliations:** 1 School of Machinery and Automation, Wuhan University of Science and Technology, Wuhan 430081, China; xuzhengqin@wust.edu.cn (Z.X.); xingyug@kotei.com (X.G.); 2 Institute of Robotics and Intelligent Systems, School of Information Science and Engineering, Wuhan University of Science and Technology, Wuhan 430081, China; 3 School of Automation Science and Engineering, South China University of Technology, Guangzhou 510640, China; zlyu@scut.edu.cn

**Keywords:** DOA estimation, RANSAC algorithm, uniform circular array, ESPRIT algorithm

## Abstract

Estimating the Direction of Arrival (DOA) is a basic and crucial problem in array signal processing. The existing DOA methods fail to obtain reliable and accurate results when noise and reverberation occur in real applications. In this paper, an accurate and robust estimation method for estimating the DOA of sources signal is proposed. Incorporating the Estimating Signal Parameters via Rotational Invariance Techniques (ESPRIT) algorithm with the RANdom SAmple Consensus (RANSAC) algorithm gives rise to the RAN-ESPRIT method, which removes outliers automatically in noise-corrupted environments. In this work, a uniform circular array (UCA) is converted into a virtual uniform linear array (ULA) to begin with. Then, the covariance matrix of the received signals of the virtual linear array is reconstructed, and the ESPRIT algorithm is deployed to estimate initial DOA of the source signal. Finally, the modified RANSAC method with automatically selected thresholds is used to fit the source signal to obtain accurate DOA. The proposed method can remove the unreliable DOA feature data and leads to more accuracy of DOA estimation of source signals in reverberation environments. Experimental results demonstrate that the proposed method is more robust and efficient compared to the traditional methods (i.e., ESPRIT, TLS-ESPRIT).

## 1. Introduction

In recent years, service robots with artificial intelligence technology have gained wide applications [1]. In order to improve the human-computer interaction service experience [2], for example, facing the person who is talking and acquiring the speech and audio signal [3], the humanoid robot is required to possess some form of accurate direction function. Since the speech signal of person is invariably a broadband signal and the room reverberation may pose a serious difficulty, the Direction of Arrival (DOA) estimation has been a very challenging task [4]. After much research efforts, various broadband signal DOA estimation algorithms have been reported, such as Incoherent Signal-subspace Method (ISM) [5], Coherent Signal-subspace Method (CSM) [6], etc. Those algorithms estimate the DOA of broadband signal, which have been processed into multigroup narrowband signals, by the DOA estimation algorithm of narrowband signal. The performance of the narrowband signal DOA estimation algorithm directly affects the accuracy of the DOA estimation of the speech signal. Therefore, accurate and robust DOA estimation of narrowband signal is of great significance for improving the intelligence of robots.

Choosing the right microphone array structure is the prerequisite for proper estimate DOA, and Uniform Linear Array (ULA) [7] and Uniform Circular Array (UCA) [8] are two commonly array structures in DOA estimation. While the ULA has the unique feature whose Vandermonde matrix form is easy to analyze and process mathematically, UCA can provide 360° information of azimuth angle with the same directional characteristics due to circular symmetry. Because the robotic application needs 360° information from sensors, we choose UCA to estimate the DOA of the source signals. Generally speaking, the classical DOA estimation techniques can be divided into two categories: the algorithms based on time difference of arrival (TDOA) [9,10,11] and the algorithms based on high-resolution spectrum estimation [12,13,14]. For the former methods, the DOA estimation of source signals is carried out by finding the time difference of the homologous signals. The main idea is to find the time difference of the homologous signals arriving in the sensors at different positions, compute the distance differences and detect the DOA of the source signals by searching for geometric knowledge. Since the small amount of calculation, good real time effect and low hardware cost, the algorithm based on TDOA has been widely used. However, there are three significant limitations of the algorithms [15], which are listed as follows: (1) Estimations of time delay and DOA are divided into two stages so the parameters used in the DOA estimation phase have been used to estimate the past time. In a sense, this algorithm is the sub-optimal DOA estimation of the source signals. (2) This algorithm is usually applied to the case with single source signals. (3) This algorithm has low accuracy in the environment contained significant noise and high reverberation [16]. By considering the limitations of the former algorithm, the algorithms based on the high-resolution spectrum estimation was proposed, which analyses the correlation matrix between the sensor signals and determine the DOA of the source signals. This technique can simultaneously estimate DOAs of multiple source signals in real time. At present, the MUltiple SIgnal Classification (MUSIC) algorithm and the Estimating Signal Parameters via Rotational Invariance Techniques (ESPRIT) algorithm [17] are two representative methods based on the high-resolution spectrum estimation. The MUSIC algorithm has a significant advantage in estimation precision, but the algorithm needs spectral peak searching and small angle search, which results in computational complexity greatly. As a typical solution, the basic idea of the ESPRIT is to estimate the signal parameters by using the rotation invariant factor and obtain the DOA estimation. The key advantage of the ESPRIT algorithm is that it eliminates the search procedure inherent in the MUSIC. Different from the MUSIC, in which the computational complexity grows exponentially, the computation grows linearly with dimension in the ESPRIT. The real-time performance, which is about computational complexity, of the ESPRIT is better than that of the MUSIC. That is the reason why we choose the ESPRIT algorithm in this paper.

With the popularization of the ESPRIT algorithm, the technique has been deeply studied by many researchers. The algorithms [18], which are based on the Total Least Squares (TLS), improve the DOA estimation accuracy, and the computation speed is also faster. The algorithm [19,20] based on the Toeplitz algorithm solves the problem of loss of array aperture caused by the ESPRIT algorithm when estimating the DOA of coherent source signals, and this algorithm improves the computation speed and accuracy of the ESPRIT. However, the real environment is complicated and often contains noise with a low signal-to-noise ratio (SNR) and reverberation, and it usually yield an arbitrary diagonal noise covariance matrix. For the ESPRIT algorithm, eigen-decomposition of the data covariance matrix does not lead to correct signal and noise subspace estimation [21]. The algorithm [22] proposed that based on two subspaces to estimate the signal and noise subspaces. However, the iterative procedure in the algorithm leads to be very time-consuming. While the diagonal elements of the covariance matrix be set as a same value, the signal and noise subspace can be directly estimated by the eigen-decomposition [23]. Another algorithm [24] proposed an iteration-free method that can offer satisfactory performance by using the matrix completion technique. But the number of resolvable sources is less than the number of sensors. Recently, a new array structure [25], the coprime array, is used to estimate DOA. Similar to the case in ULA, reverberation noise maybe leads to performance degradation of the DOA estimators in the coprime array. It is noted that there are holes in the difference coarray from a coprime array [21]. In this case, the methods based on high-resolution spectrum estimation must to use spatial smoothing to restore the rank of the virtual sensor covariance matrix and only use the consecutive lags [26]. Recently, a DOA estimation method for coprime arrays was proposed in [27], however, the method discards any non-consecutive lags and only uses the consecutive lags. Therefore, the specialty of the method [27] lead us to believe that it is unable to achieve the best estimated performance. In addition, the presence of room reverberation will lead to a certain inherent bias in DOA estimation [28], particularly, the effect of room reverberation for speech signals will be more significant [29]. Hence, further work needs to be conducted to research robust DOA estimation methods in reverberation environment. In this paper, we present a robust and accurate algorithm to estimate the source DOA in noisy and reverberant room by improving the ESPRIT algorithm with UCA. The proposed algorithm consists of the following main parts:

(1) Since the array manifold matrix of UCA does not have Vandermonde matrix structure and UCA cannot be divided into two subarrays with the rotational invariance, it is not possible to directly estimate the DOA using the ESPRIT [30]. Hence, we first convert the real UCA into the virtual ULA by using similar idea in [31,32]. The virtual ULA makes it possible to estimate DOA using the ESPRIT algorithm, which also provides 360° information with the same directional characteristics. Due to the approximation during the conversion process, the DOA estimate of source signal is not accurate at low SNR by the virtual ULA [32]. To deal with this problem, we proposed a conjugate-based method to improve the accuracy at low SNR.

(2) To further improve the performance of the DOA estimation, we propose an improved algorithm to obtain the optimal DOA estimation via the weighted average of each frame’s DOA estimation over the whole-time domain. However, under reverberant environment, only a fraction of frame’s DOA estimation is unreliable and useless. As a result, the final DOA estimation may exhibit poor accuracy and poor robustness in reverberate environment. To address the problem, we introduce the RANSAC algorithm [33] in order to improve the performance of DOA estimation algorithm. The key idea is to remove unreliable data in environment with low SNR and reverberation by using the RANSAC algorithm [34]. To the best of our knowledge, few works are reported by using the RANSAC method to remove unreliable data in DOA estimation of source signals. Its benefit is that by using simple acoustic models rather than turning to complex noise and reverberation modelling, we are still able to achieve accurate source DOA estimation in noisy environments. Although the probabilistic-based approaches [35,36,37] have been proposed to cope with DOA data in order to improve the accuracy in noisy and reverberant environments, those methods all give weights to all parameters. The weights assigned to the unreliable data are not accurate. Compared with the existing weighted averaging scheme for DOA estimation, the proposed solution removes the unreliable DOA feature data and leads to more accuracy of DOA estimation of source signals in reverberation environments. Simulation results in reverberant environments have demonstrated that the method greatly improves the robustness and accuracy of DOA estimation of source signals.

The rest of the paper is organized as follows. Section 2 describes the array structure and signal model. The details of the proposed DOA estimation method are depicted in Section 3. Section 4 presents and discusses simulation results and various simulation show that the improved method greatly improves the robustness and accuracy of DOA estimation of source signals. Finally, conclusions are drawn in the last section.

## 2. Array Structure and Signal Model

**Figure 1:**
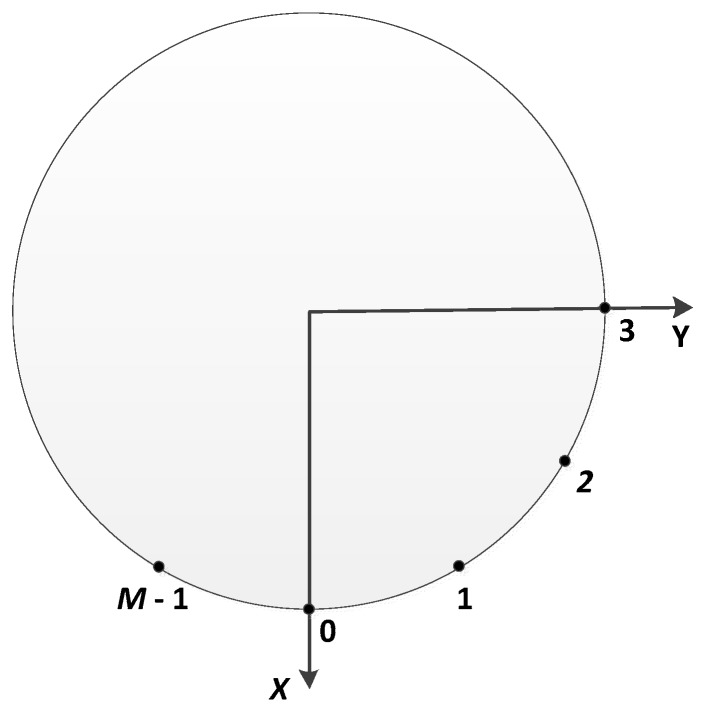
Array structure diagrammatic sketch.

UCA is considered in this work and shown in Figure 1, where identical isotropic sensors M are equally distributed on the periphery with radius of R in X-Y. A spherical coordinate system is used to indicate the direction of arrival of the incident plane wave. The origin of the coordinate system is located in the center of the array, i.e., the center of the circle. 

The source signal’s elevation angle ϕ is the angle between the Z axis and the line connecting the origin to the source signal. The source signal’s azimuth angle θ is the angle between the X axis and the projection of the connecting line on the X-Y plane. Since the DOA estimation problem of the service robots is generally only concerned with the azimuth information, it can be assumed that the elevation angle ϕ=π/2, and then, the two-dimensional DOA estimation problem is reduced to a one-dimensional DOA estimation problem [38]. If there are D uncorrelated narrowband signals, and the wavenumber is $k_{0}$, the array manifold of a UCA corresponding to the dth signal is as follows:

$$\begin{array}{c} a_{UCA}\left(\theta_{d}\right)=\left[\begin{array}{c} e^{jk_{0}Rsin\phi_{d}cos\left(\theta_{d}-\gamma_{0}\right)}\\ e^{jk_{0}Rsin\phi_{d}cos\left(\theta_{d}-\gamma_{1}\right)}\\ \vdots \\ e^{jk_{0}Rsin\phi_{d}cos\left(\theta_{d}-\gamma_{M-1}\right)} \end{array}\right] \end{array}$$

(1) $$\begin{array}{c} =\left[\begin{array}{c} e^{jk_{0}Rcos\left(\theta_{d}-\gamma_{0}\right)}\\ e^{jk_{0}Rcos\left(\theta_{d}-\gamma_{1}\right)}\\ \vdots \\ e^{jk_{0}Rcos\left(\theta_{d}-\gamma_{M-1}\right)} \end{array}\right] \end{array}$$

where $\gamma_{m}=\frac{2\pi m}{M}$, m=0,1,⋯,M−1, the second equality holds because $\phi_{d}=\pi /2$ and $\sin\phi_{d}=1$. The array manifold matrix $A_{UCA}$ of UCA is expressed as follows:

(2) $$A_{UCA}=\left[a_{UCA}\left(\theta_{1}\right),a_{UCA}\left(\theta_{2}\right),\cdots ,a_{UCA}\left(\theta_{D}\right)\right]$$

Then, the signal model received by the sensor array is:

(3) $$X_{UCA}\left(t\right)=\overset{D}{\underset{d=1}{\sum }}s_{d}\left(t\right)a_{UCA}\left(\theta_{k}\right)+N\left(t\right)=A_{UCA}S\left(t\right)+N\left(t\right)$$

where $s_{d}\left(t\right)$ is the dth signal to arrive at the sensor array, $S\left(t\right)={\left[s_{1}\left(t\right),s_{2}\left(t\right),\cdots ,s_{D}\left(t\right)\right]}^{T}$ is the signal matrix, $N\left(t\right)={\left[n_{1}\left(t\right),n_{2}\left(t\right),\cdots ,n_{M}\left(t\right)\right]}^{T}$ is an additive noise matrix.

Among the traditional DOA estimation algorithms, the high-resolution spectrum-based DOA estimation algorithms have become the one of the most common algorithms. The representative algorithms are the MUSIC and the ESPRIT. The MUSIC has high accuracy in DOA estimation, but its calculation speed is slow. The ESPRIT algorithm is faster than the MUSIC. It is important to estimate DOA of the source signal quickly in some real time applications such as robot sensing. Therefore, we choose the ESPRIT algorithm to estimate DOA of the source signals, but the accuracy of the ESPRIT in low SNR environment is low. In this paper, we propose a method to improve the accuracy of the DOA in such an environment.

## 3. Proposed DOA Estimation Method

### 3.1. Phase Mode Excitation for Circular Arrays

**Figure 2:**
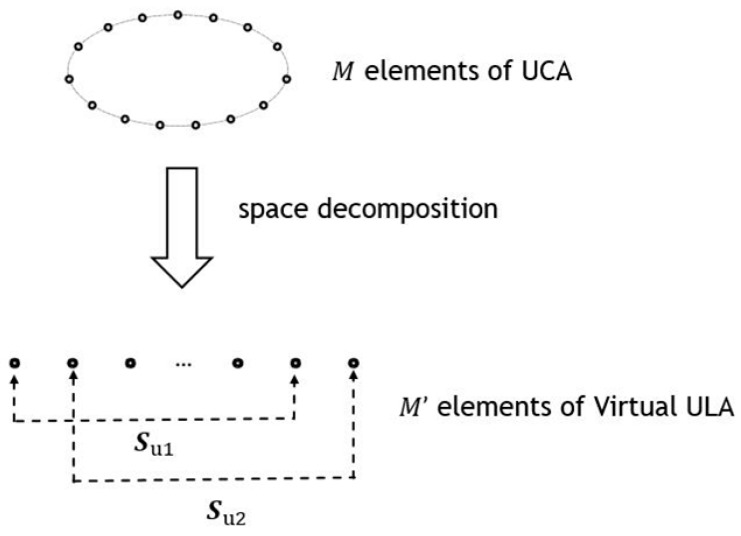
Conversion from UCA to virtual ULA.

The ESPRIT algorithm uses the translation invariance of the sensor array in the estimation of source DOAs. Unlike ULA, UCA does not have the translation invariance. Hence, the ESPRIT cannot be applied to UCA directly. To solve this problem, the idea of phase mode excitation beamformer [31] is borrowed to convert the UCA with M elements into a virtual ULA with M′ elements, as shown in Figure 2. 

For a continuous circular array, if a spatial harmonic function $\rho_{b}\left(\gamma \right)=e^{jb\gamma }$, γ∈[0, 2π] excites the aperture with phase mode b, the resulting far field pattern of the continuous circular array synthesized by the excitation function is:

(4) $$f_{b}\left(\theta \right)=\frac{1}{2\pi }\int_{0}^{2\pi }\rho_{b}\left(\gamma \right)e^{j\xi cos\left(\theta -\gamma \right)}d\gamma =j^{b}J_{b}\left(\xi \right)e^{jb\theta },$$

where $\xi =k_{0}Rsin\phi$, θ is signal’s azimuth angle and $J_{b}\left(\xi \right)$ is the Bessel function of the first kind of order b, and $J_{b}\left(\xi \right)\approx 0$ when b>ξ. Hence, the maximum mode excited by the continuous circular array is $B\approx k_{0}R$ (B is an integer). The total number of the modes excited by the continuous circular array is $M^{\prime }=2B+1$. 

The discrete UCA shown in Figure 1 can be considered as sampled uniformly from the continuous circular array. Sampling the excitation function $\rho_{b}\left(\gamma \right)$ at the array element locations results in the phase mode excitation beamforming weight vector:

(5) $$w_{b}^{H}=\frac{1}{M}\left[e^{jb\gamma_{0}},\;e^{jb\gamma_{1}},\;\cdots ,\;e^{jb\gamma_{M-1}}\right],$$

where $\gamma_{m}=\frac{2\pi m}{M}$. The resulting UCA far field pattern for mode b is:

$$\begin{array}{c} P_{b}\left(\theta \right)=w_{b}^{H}a_{UCA}\left(\theta \right)=\frac{1}{M}\overset{M-1}{\underset{m=0}{\sum }}e^{jb\gamma_{m}}e^{j\xi cos\left(\theta -\gamma_{m}\right)} \end{array}$$

(6) $$\begin{array}{c} =j^{b}J_{b}\left(\xi \right)e^{jb\theta }+\overset{\infty }{\underset{i\neq 0}{\sum }}\left(j^{g}J_{g}\left(\xi \right)e^{-jg\theta }+j^{h}J_{h}\left(\xi \right)e^{-jh\theta }\right), \end{array}$$

where g=Mi−b and h=Mi+b. The terms under the summation in Equation (6), which cause the discrete UCA pattern to deviate from that of the continuous circular array, can be made negligible by choosing M to be sufficiently large. Hence, by selecting sufficiently large M, the UCA far field pattern for mode b is approximated as:

(7) $$l_{b}\left(\phi ,\;\theta \right)\approx j^{b}J_{b}\left(\xi \right)e^{jb\theta },\;\left|b\right|\leq B$$

As all the modes are excited with reasonable strength, here we adopt the phase mode excitation beamformer $F_{u}^{H}=C_{u}V^{H}$, where

(8) $$\{\begin{array}{l} C_{u}=diag\left\{j^{B},\;\cdots ,j^{1},\;j^{0},\;j^{-1},\;\cdots ,\;j^{-B}\right\}\\ V^{H}=\sqrt{M}{\left[w_{-B},\;\cdots ,\;w_{0},\;\cdots ,\;w_{B}\right]}^{H} \end{array}$$

and the resulting manifold of the $M^{\prime }=2B+1$ dimensional beamspace synthesized by the orthogonal beamformer $F_{u}^{H}$ is:

(9) $$a_{u}\left(\theta_{d}\right)=F_{u}^{H}a_{UCA}\left(\theta_{d}\right)=\left[\begin{array}{c} J_{-B}\left(\xi \right)e^{-jB\theta_{d}}\\ \vdots \\ J_{-1}\left(\xi \right)e^{-j\theta_{d}}\\ J_{0}\left(\xi \right)\\ J_{1}\left(\xi \right)e^{j\theta_{d}}\\ \vdots \\ J_{B}\left(\xi \right)e^{jB\theta_{d}} \end{array}\right]$$

From the condition in Equation (1), we can observe that Bessel function terms in Equation (9) are constants and can be calculated directly. Hence, a new direction vector can be obtained by a weight matrix that eliminates the Bessel function terms. The weight matrix is:

(10) $$J_{\varsigma }=diag\left\{J_{-B}^{-1}\left(\varsigma \right),\cdots ,J_{1}^{-1}\left(\varsigma \right),J_{0}^{-1}\left(\varsigma \right),J_{1}^{-1}\left(\varsigma \right),\cdots ,J_{B}^{-1}\left(\varsigma \right)\right\}$$

where $\varsigma =k_{0}R$. The resulting new direction vector is

(11) $$a\left(\theta_{d}\right)={\left[e^{-jB\theta_{d}},\cdots ,e^{j\theta_{d}},0,e^{j\theta_{d}},\cdots ,e^{jB\theta_{d}}\right]}^{T}$$

Hence, the resulting virtual ULA output signal is

(12) $$X\left(t\right)=J_{\varsigma }F_{u}^{H}X_{UCA}\left(t\right)=AS\left(t\right)+N_{u}\left(t\right)$$

where $A=J_{\varsigma }F_{u}^{H}A_{UCA}$, $N_{u}\left(t\right)=J_{\varsigma }F_{u}^{H}N\left(t\right)$. And the DOA estimation can be obtained by the ESPRIT algorithm, which is described in the next section.

From Equation (12), the beam space covariance matrix is:

(13) $$R_{x}=E\left[X\left(t\right)X^{H}\left(t\right)\right]=AR_{s}A^{H}+R_{F}$$

where $R_{s}$ is the covariance matrix of S(t), $R_{F}$ is the covariance matrix of $JF_{u}^{H}N\left(t\right)$. Thus, we can get matrix S that spans signal subspace of $R_{x}$. The terms under the summation can be neglected in Equation (6). Here, we compare the DOA estimation accuracy of a virtual ULA and the real ULA, which has the same sensor number and spacing as the virtual ULA.

**Figure 3:**
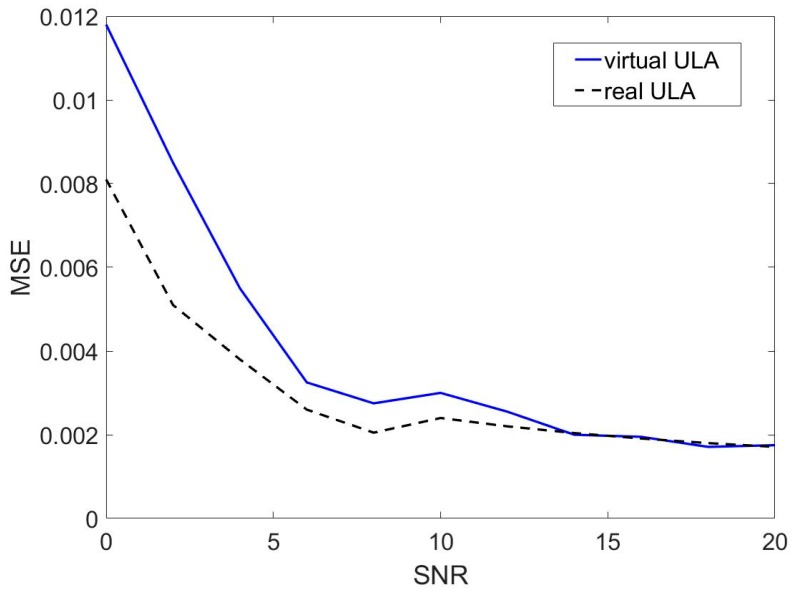
MSEs of virtual ULA and real ULA.

Assuming that the sixteen sensors on the UCA are evenly distributed on the circumference with R=λ/2 and a source signal is incident on the array at 40°. The noise is composed of Gaussian noise. The Gaussian noise follows a normal distribution with mean value 0 and standard deviations σ. The signal with different SNR is obtained by artificially adding Gaussian noise. The simulations are based on 100 Monte Carlo. We define the MSE as $\mathrm{MSE}=\frac{1}{n}\sum_{a}^{n}{\left(\theta_{a}-\theta_{t}\right)}^{2}$, where n=100, $\theta_{a}$ is the ath Monte Carlo DOA estimation and $\theta_{t}$ is the ground truth DOA. It can be seen from Figure 3 that there is good agreement between the virtual ULA and the real ULA at SNR > 10 dB. However, there exists the deviation between the virtual ULA and the real ULA at 10 dB > SNR > 0 dB and the deviation is the maximum at SNR = 0 dB. Hence, the change of deviation between the virtual ULA and the real ULA verifies the fact that the approximation employed in Equation (6) is not accurate enough at this low SNR.

### 3.2. Reconstruction of the Covariance Matrix

To improve DOA estimations of source signals at low SNR, the idea of the combination of the ordinary and the conjugate data [39,40] is borrowed to obtain more accurate DOA estimations. 

The conjugate form of Equation (12) is:

(14) $$\bar{X}\left(t\right)=\bar{A}\bar{S}\left(t\right)+{\bar{N}}_{u}\left(t\right)$$

where the overbar denotes the conjugate of each element of a vector or of a matrix. An improved DOA estimation can be obtained by using the combined information of X(t) and $\bar{X}\left(t\right)$. Let J be an exchange matrix expressed by:

(15) $$J=\left[\begin{array}{ccccc} 0 & 0 & \cdots & 0 & 1\\ 0 & 0 & \cdots & 1 & 0\\ \vdots & \vdots & \ddots & \vdots & \vdots \\ 0 & 1 & \cdots & 0 & 0\\ 1 & 0 & \cdots & 0 & 0 \end{array}\right]$$

Here, J is a symmetric matrix and $J^{2}=I$ where I is an identity matrix. Hence, the resulting covariance matrix $R_{\bar{x}}$ of $J\bar{X}\left(t\right)$ is:

(16) $$R_{\bar{x}}=E\left[J\bar{X}\left(t\right){\bar{X}}^{H}\left(t\right)J\right]=J\bar{A}{\bar{R}}_{s}{\bar{A}}^{H}J+J{\bar{R}}_{F}J=J{\bar{R}}_{x}J$$

We define the matrix $R_{\bar{x}x}$ as:

(17) $$R_{\bar{x}x}=R_{x}+R_{\bar{x}}=R_{x}+J{\bar{R}}_{x}J$$

The signal subspace of $AR_{s}A^{H}$ and $J\bar{A}{\bar{R}}_{s}{\bar{A}}^{H}J$ and $AR_{s}A^{H}+J\bar{A}{\bar{R}}_{s}{\bar{A}}^{H}J$ are all the same (see Lemma A1 in Appendix A for details). Hence, by using SVD of $R_{\bar{x}x}$, the signal subspace matrix ***S*** = AT, where T is a nonsingular matrix. Let $S_{u1}$ and $S_{u2}$ shown in Figure 2 be the matrices that respectively pick out the first and last $M_{e}=M^{\prime }-1$ elements from $S_{u}\;$. The resulting relationship between $S_{u1}$ and $S_{u2}$ is:

(18) $$S_{u2}=S_{u1}\Psi \Rightarrow S_{u1}^{+}S_{u2}=\Psi$$

where ‘+’ denotes the generalized inverse of the matrix and $\Psi =T^{-1}\Phi T$. Hence, the rotation invariant relation matrix Φ between $S_{u1}\;\mathrm{and}\;S_{u2}$ is:

(19) $$\Phi =T\Psi T^{-1}=diag\left[e^{j\theta_{1}},e^{j\theta_{2}},\cdots ,e^{j\theta_{D}}\right]$$

**Figure 4:**
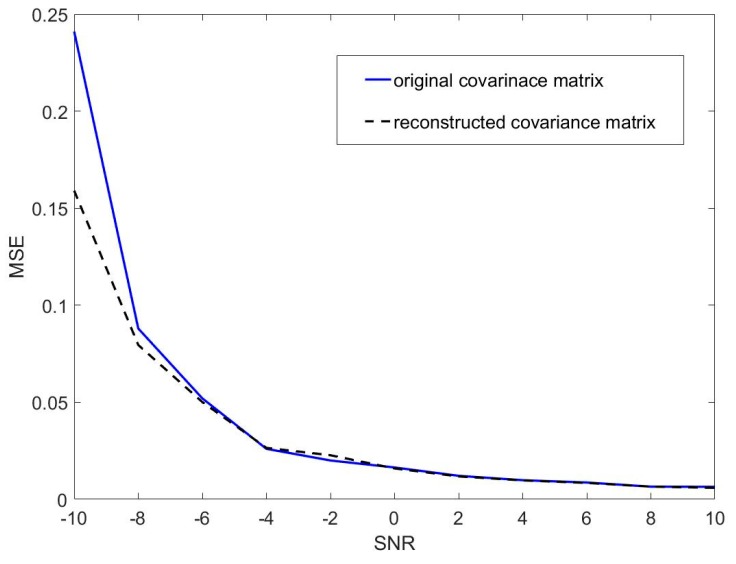
MSEs of the original covariance matrix $R_{x}$ and the reconstructed covariance matrix $R_{\bar{x}x}$ in the environment contained noise.

**Figure 5:**
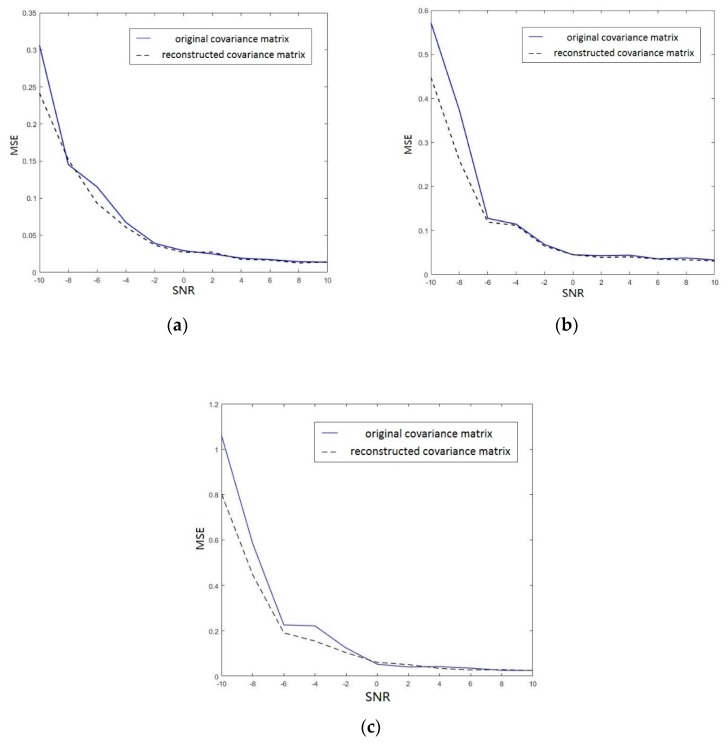
MSEs of the original covariance matrix $R_{x}$ and the reconstructed covariance matrix $R_{\bar{x}x}$ in the environment contained noise and reverberation. (**a**) the sound reflection coefficient is 0.15 (RT60 = 100 ms); (**b**) the sound reflection coefficient is 0.2; and (**c**) the sound reflection coefficient is 0.25.

The diagonal elements of Φ are the eigenvalues of Ψ given by $\mu_{k}=e^{\theta_{k}}$, k=1,⋯,D. The resulting azimuth angle $\theta_{k}$ of the kth source signal is $\theta_{k}=arg\left(\mu_{k}\right)$. And it is strongly consistent with the true azimuth angle of source signal (see Lemma A2 in the Appendix A for the details). Then, we compare the accuracies of DOA estimations based original covariance matrix $R_{x}$ and reconstructed covariance matrix $R_{\bar{x}x}$, respectively. The results on condition that there is Gaussian noise without reverberation are shown in Figure 4. Then, we use the RoomSim [41] to simulate a virtual room (RT60 = 100 ms), and obtain reverberation of different intensities by changing the reflection coefficient. The results are shown in Figure 5.

Since the environment contains noise, it can be seen from Figure 4 that the accuracy of the reconstructed covariance matrix is almost the same with the original covariance matrix at SNR > −8 dB, but the former is significantly higher than the latter at SNR = −10 dB; With the change of the sound reflection coefficient, the different reverberation environment can be constructed. Furthermore, the bigger the sound reflection coefficient is, the stronger the reverberation in the environment is. It is shown in Figure 5a–c that the accuracy of the reconstructed covariance matrix is almost the same with the original covariance matrix at high SNR, but the former is significantly higher than the latter at low SNR. And the difference between the reconstructed covariance matrix and the original covariance matrix become clearer as the reverberation increases. Hence, it is clear for all to see that the reconstructed covariance matrix effectively improves accuracy of DOA estimation at low SNR with both noise and reverberation.

### 3.3. DOA Estimation Based on RANSAC

The source signals often contain noises and reverberation in real environments, the subspace S obtained by SVD of $R_{\bar{x}x}$ are thus polluted. Hence, the resulting DOA estimations of source signals are not accurate. To improve the accuracy of the DOA, we divide the source signals into N frames and estimate the DOA of each frame. Then, we can obtain accurate DOA estimations by fitting the DOA estimations in all frames.

Before we start to fit the DOAs, we observe that for multi-source signals, the same signal may be spread into different positions in Φ of different frames because of the influence of noise and reverberation. To solve the problem, the uncorrelation between different source signals in $S_{u}$ is used to match the DOA of the same source signal in different frames. 

Generally, sound signals vary over time but they can be viewed as stationary in a short period of time, say within 10–30 ms [42]. In this study, when sampling frequency is 44 kHz and the signals are obtained by adding the Hamming window, which is an overlapping windows and the overlap is 50%, with the length of 256, the obtained every 50 frames signals are regarded as stationary signals which update every 14.5 ms. The DOA is calculated for 50 segments each having 256 samples. To avoid a delay before getting the first DOA estimate, we use zero padding method to process the start of the signal. Let $s_{d,1}\left(t\right),\cdots ,s_{d,N}\left(t\right)$ be the dth source signal in N frames, and the first frame and $s_{d,1}\left(t\right)$ be the reference frame and the *d*th reference signal, respectively. The source signal $s_{d,n}\left(t\right)$, n=2,⋯,N, has the same characteristic with the reference signal. According to Equation (19), T is the eigenvector matrix of Ψ. The eigenvectors that correspond to the same source signal in different frames’ T are correlated based on the characteristic of stationary signal. $T_{1}$ and $T_{i}$, i=2,⋯,N, are the eigenvector matrices of Ψ in the reference frame and the ith frame, respectively. We introduce the correlation matrix:

(20) $$G_{i}=T_{1}^{H}T_{i}$$

The eigenvectors that correspond to the same source signal in $T_{1}$ and $T_{i}$ are maximum correlation. Hence, the resulting matrix coordinate $g_{uv}$ of the largest element of each column in $G_{i}$, which represents that the uth DOA in the reference frame and the vth DOA in the ith frame, are the DOA of the same source signal. In this way, we can obtain D groups of parameters for DOA estimation that have been matched by rearranging the parameters in each frame. Then the optimal DOAs of D source signals can be obtained in next section.

Because in the environments with noise and reverberation, the DOA estimation may be abrupt at low SNR. Straightforward usage of these DOAs may be not accurate enough. To solve the problem, the idea of RANSAC [33] is borrowed to exclude the unreliable parameter. The RANSAC algorithm is a very robust algorithm to estimate DOA while removing the unreliable DOA feature data (noisy and inaccurate values). Hence, we can obtain the optimal DOA estimation of the D source signals by RANSAC.

For N frames, we compute the DOA of each source and match them. We can obtain N groups of DOA parameters that have been matched. Then some iterations are performed to achieve the optimal DOA set without unreliable DOA feature data and further obtain optimal DOA estimation by fitting the set, where the number of iterations h is defined in [33] by:

(21) $$h=\frac{\log\left(1-p\right)}{\log\left(1-w^{f}\right)}$$

where p is the probability, w is the proportion of inliers, and f is the number of randomly selected DOAs. There may be no upper bound on the time that RANSAC takes to converge. When one increases the number h of RANSAC iteration, the probability to the optimal DOA increase, but the computational cost increases as well. In the paper, we set the iteration number h=50 as a trade-off between the optimal DOA and the computational load.

In each RANSAC iteration, for each group DOA, f parameters for random sampling are needed. Subsequently, the linear equation is adopted to calculate the primary curve corresponding to the DOA. If the parameter of each frame is exactly on the primary curve, the DOA feature data is accurate. Thus, the distance l between the parameter of each frame and the primary curve determines the accuracy DOA. The distance of ith parameter is defined as:

(22) $$l_{i}=\left|\theta_{i}-\bar{\theta }\right|$$

where $\bar{\theta }=\theta_{1}k_{1}+\theta_{2}k_{2}+\ldots +\theta_{N}k_{N}$, $k_{i}=\frac{1}{\sqrt{2\pi }\delta }e^{-\frac{{\left(\theta_{i}-\mu \right)}^{2}}{2\delta^{2}}}$, δ and μ are the variance and the expectation of *N* frames DOAs.

After the distance is calculated, we should consider how to choose the distance threshold ε. If the distance between the parameter of each frame and the primary curve is larger than ε, the corresponding parameter is identified as outlier and will be removed in the next step. It is difficult for us to choose the distance threshold ε. A very high threshold may mistakenly classify the outliers into the consensus set while a very low threshold may cause instability in some case because of unreliable DOA feature data. In this paper, the threshold ε is selected by the distance in Equation (22). Let $l_{i}$ be arranged in a descending order, and the δNth (if δN is not an integer, round towards plus infinity) is the threshold value ε. This rule for selecting the threshold is also applicable to other situations. For the different N frames’ DOAs, the method can select the appropriate threshold value ε and effectively avoid the influence of the positioning result caused by improper selection of the threshold.

**Figure 6:**
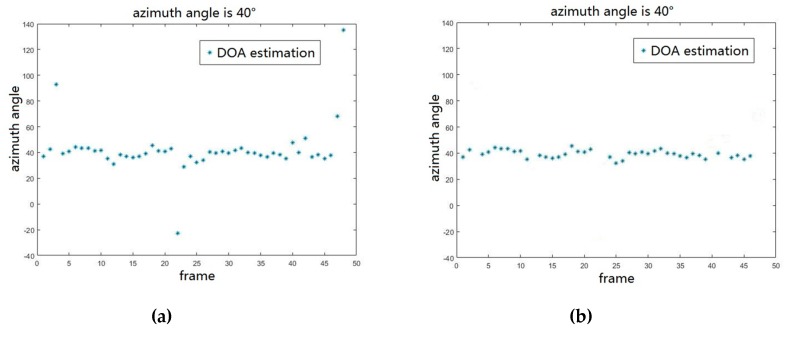
(**a**). the multiply frames’ DOA estimations. (**b**). the multiply frames’ DOA estimations processed by RANSAC algorithm.

The resulting largest consensus set that contains the most exact DOA parameter is obtained and the DOAs without unreliable parameters are determined. Assuming that there are sixteen sensors on the UCA are evenly distributed on the circumference with R=λ/2, one source signal is incident on the array at 40° and SNR=−15 dB. In this study, we use the RoomSim to simulate a virtual room (RT60 = 100 ms). The resulting multiple frames’ signal DOAs obtained by RANSAC are shown in Figure 6.

In this simulation, low SNR environment with reverberation is considered. It can be seen from the results shown in Figure 6a that the DOA estimations in most of frames have good consistency, but the DOA estimations influenced by the low SNR and reverberation become unreliable in a few frames. From the results in Figure 6b, the unreliable DOA that is shown in Figure 6a have been removed by the RANSAC. It means that the more accurate DOA estimation can be obtained by fitting. Hence, the optimal DOA of a source signal can be obtained by fitting the DOAs. In this way, we can obtain the optimal DOAs of D source signals.

In summary, the RAN-ESPRIT algorithm is described as follows:
**Algorithm 1**: RAN-ESPRIT algorithm(1)Take multiple frames of source signals and perform step (2)–(6) for each frame;(2)Convert the UCA output signal to virtual ULA by Equation (12), and form the virtual ULA covariance matrix $R_{x}=E\left[X^{H}X\right]$;(3)Reconstruct the matrix $R_{\bar{x}x}$ by Equation (17);(4)Obtain the signal subspace matrix S by SVD of $R_{\bar{x}x}$;(5)Partition S into $S_{u1}$ and $S_{u2}$, and obtain Ψ by Equation (18);(6)Compute the eigenvalues $\mu_{d}=e^{\theta_{d}}$, d=1,⋯,D of Ψ. The estimate of azimuth angle $\theta_{d}$ of the dth source signals is $\theta_{d}=arg\left(\mu_{d}\right)$;(7)Match the DOA of each source signal in each frame to get D groups of DOA by Equation (20). Each group contains the DOA of the same source signals in N frames. Perform step (8)–(13) for each group;(8)Set parameters for adaptive RANSAC method: h=∞,ε=δN,i=1;(9)Randomly select f DOAs of the group and calculate the primary curve by the linear equation;(10)Identify the threshold value ε and a set of inliers consistent with the primary curve;(11)Update the number of iterations h by Equation (21), if a larger consistent set is found;(12)If i>h, the largest consensus set is acquired, go to step (13); else i=i+1, go to step (7);(13)Once the largest consensus is obtained, the subset of DOA of the group is determined. The optimal DOAs of each source signal are obtained by fitting the subset in each group.

## 4. Experimental Results and Discussions

### 4.1. Experimental Results

Simulations have been performed to verify and analyze the proposed method in this section. The sixteen sensors on the UCA are evenly distributed on the circumference with parameter R=λ/2. Two uncorrelated narrowband source signals are incident on the array at 40° and 80° respectively. The two signals in each frame are obtained by adding the Hamming window with the length of 256. To verify the robustness of the proposed method, we inject Gaussian noise which follows a normal distribution with mean value 0 and standard deviations σ. The signals in different SNRs are imported into a virtual indoor room to build reverberation. the RoomSim is employed to simulate a virtual room (RT60 = 100 ms), and reverberations in different intensities are generated by changing the reflection coefficients. The UCA is located at the center of the virtual room. The simulations are based on 100 Monte Carlo runs.

In this section, four methods are analyzed: (1) the proposed algorithm in this paper (denoted by Line 1); (2) UCA-ESPRIT algorithm [32] is fitted by the least square (LS) method (denoted by Line 2); (3) The algorithm [18] is fitted by the LS method (denoted by Line 3); (4) The algorithm [19] is fitted by the LS method (denoted by Line 4); Three individual simulations are conducted. The first tests the DOA estimation accuracy at different SNR environment with noise only, the second tests the DOA accuracy of different coherent noise level and the last tests the DOA accuracy of different SNR environments with Gaussian noise and coherent noise.

It can be seen from the experimental results shown in Table 1 that the Line 1 is significantly better than other three method in the two environments that contained only noise and contained both noise and reverberation. It is clear that the difference of Line 1 is the least in the two environments. It means that the method of this paper is quite efficient to improve DOA estimation accuracy compared to the traditional methods. 

**Figure 7:**
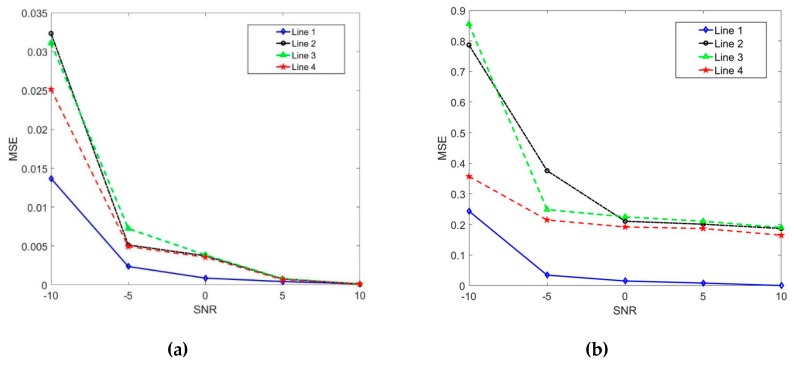
(**a**). MSEs of the four methods at −10 dB < SNR < 10 dB (without reverberation). (**b**). MSEs of the four methods at −10 dB < SNR < 10 dB (contain reverberation and the sound reflection coefficient is 0.25).

Moreover, the experimental results for different SNR (without reverberation) have been reported in Figure 7a. It can be seen that the accuracy of DOA estimation with the Line 1 is obviously higher than the others. From Figure 7a shows that the performance of Line 1 is obviously higher than the others. Hence, in the case of the different SNR (without reverberation), it is clear to see that the robustness and accuracy of the DOA estimation have been improved by the proposed algorithm in this paper. 

In the virtual space, the performance of four methods at different SNR (contain reverberation) is compared and the results are shown in Figure 7b. It can be seen that the DOA estimation accuracy of the Line 1 is obviously higher than the others. In Figure 7b, the performance of Line 1 is obviously higher than the others. Hence, it is clear that the accuracy of the Line 1 is less affected by the reverberation at the same SNR. That is to say, the robustness and accuracy of the DOA estimation have been improved by the presented algorithm in this paper. In summary, the above three experiments demonstrate that the proposed algorithm can not only produce high accuracy for DOA feature data at the same input noise level, but also can be more robust, regardless of whether the environment contains reverberation or not.

### 4.2. Discussion

In this section we discuss the estimation procedure of the DOA of source signal in the environment contained reverberation. In order to obtain 360° information from the sensor arrays, we choose the UCA to estimate DOA. Liu [32] proposes a technique to convert the UCA into a virtual ULA. It makes the ESPRIT algorithm effective in UCA. In Section 3.1, we describe the conversion process in detail. And from Figure 3, it is obvious that the virtual ULA is not accurate enough at low SNR.

In Section 3.2, we proposed a modified method to improve the accuracy of DOA estimation and verify the consistency of the modified method. Kundu [38,39] proposes a method to obtain the more accurate DOA estimation by using conjugate matrix. It performs better than the previously existing methods. The solution depends on the roots in a certain symmetry. We observe that the coefficients of the polynomial exhibit a certain symmetry. It is well known that the constrained estimates have lower variance than the unconstrained one. Now we give some justifications to enhance the superiority of the modified method. We have already seen that the null space of $AR_{s}A^{H}$ and $AR_{s}A^{H}+J\bar{A}{\bar{R}}_{s}{\bar{A}}^{H}J$ are the same. The basic idea of the modified method is to obtain vectors of the form $a\left(\theta_{d}\right)$ (as defined in Equation (11)) that are orthogonal to the corresponding null spaces. So naturally the performance of the methods depends on how good the approximation of the null space is, which is made from the given data. If we can know exactly $R_{\bar{x}x}$, then we can estimate the DOAs without any error. Since the matrix $R_{\bar{x}x}$ is unknown so it is estimated by ${\hat{R}}_{\bar{x}x}$, where is the sample estimate of $R_{\bar{x}x}$. Observe that as the length of the signals tends to infinity then by the law of large numbers, the ${\hat{R}}_{\bar{x}x}$ converge to $R_{\bar{x}x}$, which follows from the law of iterated logarithm. And it is interesting to observe that both ${\hat{R}}_{\bar{x}x}$ and $R_{\bar{x}x}$ satisfy a conjugate symmetry constraint, namely ${\hat{R}}_{\bar{x}x}=J{\hat{R}}_{\bar{x}x}J$ and $R_{\bar{x}x}=J{\bar{R}}_{\bar{x}x}J$. It is well known that if constrained estimates are used then it produces better results than the ordinary estimates for finite sample. The results shown in Figure 4; Figure 5 confirm the higher accuracy of the modified method.

From Figure 6a, it is obvious that the DOA estimation becomes unreliable at low SNR with reverberation. We find the RANSAC algorithm is quite satisfactory when dealing with data outliers. In Section 3.3, we propose a DOA estimation based RANSAC. The algorithm iteratively seeks the optimal persistent set, which has the maximal number of inliers by leaving out the outliers. Using the inliers, the final source signal’s DOA is estimated. The advantage of RANSAC is that it does not require explicitly modelling of noise or reverberation, while obtaining the accurate DOA estimation in adverse environments. From Figure 6b, it is obvious that data outliers (polluted DOAs) have been left out effectively. It is observed from Figure 7 that the proposed algorithm is very robust and accurate.

Finally, we discuss the effects of the threshold used in RANSAC algorithm. While being very robust in dealing with data outliers, the RANSAC algorithm is affected by the threshold and is unstable. Hence it is important to choose the threshold appropriately. If a very high threshold is chosen, it is possible that we may mistakenly classify the outliers into the consensus set, and the accuracy of RANSAC decreases. If a very low threshold is chosen, it is possible that we are not able to get the optimal set. Comparing to Figure 7, it is observed that the threshold selection method is satisfactory.

## 5. Conclusions

In this paper, we converted the UCA into a virtual ULA and presented a modified method based conjugate matrix to improve accuracy of the conversion. Then, we proposed an accurate and reliable technique to estimate DOA of source signals based on RANSAC algorithm. Experimental results also show that the proposed approach is robust and efficient in the environments with noise and reverberation when compared with the other existing methods. In the real environment, there is a larger variety of source signals, with sources at a variety of distance from the array. We will conduct further research on this issue in our future work.

## Figures and Tables

**Table 1:** Comparative results of DOA (40°).

Method	SNR=−15 dB	SNR=−15 dB (Contains Reverberation)
Line 1	40.8530	41.1326
Line 2	35.8619	33.1799
Line 3	37.7511	34.0132
Line 4	37.6058	43.3901

## Appendix A

**Lemma** **A1.**
*The signal subspaces of $AR_{s}A^{H}$ and $J\bar{A}{\bar{R}}_{s}{\bar{A}}^{H}J$ and $AR_{s}A^{H}+J\bar{A}{\bar{R}}_{s}{\bar{A}}^{H}J$ are all the same.*

**Proof.** It follows from [43] that for any $\theta_{1},\cdots ,\theta_{D}$, there exists $g_{1},g_{2},\cdots ,g_{M\prime }$ such that $z_{1}=e^{j\theta_{1}},\cdots ,z_{D}=e^{j\theta_{D}}$ are the roots of the following polynomial:

(A1) $$P\left(z\right)=g_{1}z^{-B}+\cdots +g_{B}z^{-1}+g_{B+2}z^{1}+\cdots +g_{M^{\prime }}z^{B}$$

Since the roots of polynomial P(z) are of unit modulus, we have:

(A2) $${\bar{z}}_{d}=z_{d}^{-1},\;d=1,2,\cdots ,D$$

We introduce a new polynomial Q(z) as follows:

(A3) $$Q\left(z\right)={\bar{g}}_{1}z^{B}+\cdots +{\bar{g}}_{B}z^{1}+{\bar{g}}_{B+2}z^{-1}+\cdots +{\bar{g}}_{M\prime }z^{-B}$$

Equations (A2) and (A3) have the same roots. Comparing the coefficients of the two polynomials, we can obtain:

(A4) $$\frac{g_{i}}{{\bar{g}}_{M\prime -i}}=\frac{g_{M\prime }}{{\bar{g}}_{1}},\;i=1,2,\cdots ,M^{\prime }$$

We define:

(A5) $$b_{i}=g_{i}{\left({\bar{g}}_{1}/g_{M\prime }\right)}^{1/2}$$

We have $b_{i}={\bar{b}}_{M\prime -i}$, it can be written in the form of matrix as follows:

(A6) $$b=J\bar{b}$$

where $b={\left[b_{0},b_{1},\cdots ,b_{D}\right]}^{T}$, J is the exchange matrix defined in Equation (15). Let $u=\left(u_{1},u_{2},\cdots ,u_{M\prime }\right)$ be an eigenvector of $AR_{s}A^{H}$ corresponding to the zero eigenvalue. Since $rank\left\{R_{s}\right\}=D$, $AR_{s}A^{H}u=0$ if and only if $A^{H}u=0$, then:

(A7) $$u_{1}e^{-jB\theta_{d}}+\cdots +u_{B}e^{-j\theta_{d}}+u_{B+2}e^{j\theta_{d}}+\cdots +u_{M\prime }e^{jB\theta_{d}}=0$$

Now we have:

(A8) $$u_{M\prime }e^{-jB\theta_{d}}+\cdots +u_{B+2}e^{-j\theta_{d}}+u_{B}e^{j\theta_{d}}+\cdots +u_{1}e^{jB\theta_{d}}=0$$

The resulting Equation (A8) is equivalent to ${\bar{A}}^{H}Ju=0$, which implies $J\bar{A}{\bar{R}}_{s}{\bar{A}}^{H}Ju=0$. From Equations (A6)–(A8), it is obvious that for any u, there exists a non-zero complex constant c such that:

(A9) $$b=cu,\;\mathrm{and}\;b=J\bar{b}$$

Hence:

$$\begin{array}{c} A^{H}u=0⟹A^{H}b=0 \end{array}$$

$$\begin{array}{c} ⟹J\bar{A}{\bar{R}}_{s}{\bar{A}}^{H}JJ\bar{b}=0\;\mathrm{since}\;J^{2}=I \end{array}$$

(A10) $$\begin{array}{c} ⟹J\bar{A}{\bar{R}}_{s}{\bar{A}}^{H}Ju=0 \end{array}$$

Given as such, the resulting null space of $AR_{s}A^{H}$, $J\bar{A}{\bar{R}}_{s}{\bar{A}}^{H}J$ and $AR_{s}A^{H}+J\bar{A}{\bar{R}}_{s}{\bar{A}}^{H}J$ are all the same. Therefore, we can further conclude that the signal subspace of $AR_{s}A^{H}$ and $J\bar{A}{\bar{R}}_{s}{\bar{A}}^{H}J$ and $AR_{s}A^{H}+J\bar{A}{\bar{R}}_{s}{\bar{A}}^{H}J$ are also the same.

**Lemma** **A2.**
*The DOA estimations based on the modified covariance matrix are strongly consistent with the true DOAs of source signals.*

**Proof.** The eigenvalues of $AR_{s}A^{H}$ satisfy the following inequality:

(A11) $$\lambda_{1}\geq \lambda_{2}\cdots \geq \lambda_{D}\geq \lambda_{D+1}=\cdots =\lambda_{M^{\prime }}$$

Let $e_{1},\cdots ,e_{M\prime }$ be the corresponding normalized orthogonal eigenvectors, we define:

(A12) $$E_{s}=\left(e_{1},\cdots ,e_{D}\right),\;\;E_{n}=\left(e_{D+1},\cdots ,e_{M\prime }\right)$$

The eigenvalues of $AR_{s}A^{H}+J\bar{A}{\bar{R}}_{s}{\bar{A}}^{H}J$ satisfy:

(A13) $$\mu_{1}\geq \mu_{2}\cdots \geq \mu_{D}\geq \mu_{D+1}=\cdots =\mu_{M^{\prime }}$$

Let $w_{1},\cdots ,w_{M\prime }$ be the corresponding normalized orthogonal eigenvectors, we define:

(A14) $$U_{s}=\left(w_{1},\cdots ,w_{D}\right),\;\;U_{n}=\left(w_{D+1},\cdots ,w_{M\prime }\right)$$

Let ${\hat{\lambda }}_{1}\geq {\hat{\lambda }}_{2}\cdots \geq {\hat{\lambda }}_{M\prime }$ be the eigenvalues of $X\left(t\right)X^{H}\left(t\right)$ and let ${\hat{e}}_{1},\cdots ,{\hat{e}}_{M\prime }$ be the corresponding normalized orthogonal eigenvectors. Similarly Let ${\hat{\mu }}_{1}\geq {\hat{\mu }}_{2}\cdots \geq {\hat{\mu }}_{M\prime }$ be the eigenvalues of $X\left(t\right)X^{H}\left(t\right)+J\bar{X}\left(t\right){\bar{X}}^{H}\left(t\right)J$ and let ${\hat{e}}_{1},\cdots ,{\hat{e}}_{M\prime }$ be the corresponding normalized orthogonal eigenvectors. It is easy to obtain that:

(A15) $$a^{T}\left(\theta_{d}\right)E_{n}=0\Leftrightarrow C\left(\theta_{d}\right)=a^{T}\left(\theta_{d}\right)E_{n}E_{n}^{H}\bar{a}\left(\theta_{d}\right)=0$$

and:

(A16) $$a^{T}\left(\theta_{d}\right)U_{n}=0\Leftrightarrow D\left(\theta_{d}\right)=a^{T}\left(\theta_{d}\right)U_{n}U_{n}^{H}\bar{a}\left(\theta_{d}\right)=0$$

where $a\left(\theta_{d}\right)$, d=1,2,⋯,D, are defined in Equation (11). However, $E_{n}$ and $U_{n}$ are unknown in practice, we only can take ${\hat{E}}_{n}=\left({\hat{e}}_{D+1},\cdots ,{\hat{e}}_{M\prime }\right)$ and ${\hat{U}}_{n}=\left({\hat{w}}_{D+1},\cdots ,{\hat{w}}_{M\prime }\right)$ to be the estimates of $E_{n}$ and $U_{n}$, respectively. Along with the idea in [44], we can obtain the coefficients ${\hat{b}}_{1},{\hat{b}}_{2},\;\cdots ,\;{\hat{b}}_{M\prime }$ by using the elements in ${\hat{E}}_{n}$ and ${\hat{U}}_{n}$ first, and then we can estimate $e^{j\theta_{d}}$ by solving:

(A17) $$B\left(z\right)={\hat{b}}_{1}z^{-B}+\cdots +{\hat{b}}_{B}z^{-1}+0+{\hat{b}}_{B+2}z^{1}+\cdots +{\hat{b}}_{M\prime }z^{B}=0$$

The coefficients are chosen to hold the same constraints as (A6). The constrained estimators are more efficient than the unconstrained one. Hence, the resulting estimations obtained by Equation (A17) are strongly consistent with the true DOAs of source signals.
